# Gram-negative Cranial Bone Flap Infection Treated with Continuous Gentamicin Irrigation: A Case Report

**DOI:** 10.7759/cureus.4282

**Published:** 2019-03-20

**Authors:** Jamie Toms, Lisa Kurczewski, Robert Simonds, R. Scott Graham, Jason Harrison

**Affiliations:** 1 Neurosurgery, Virginia Commonwealth University Health Systems, Richmond, USA; 2 Neurosurgery, Harbin Clinic, Rome, USA

**Keywords:** continuous irrigation, bone flap infection, craniotomy, subdural empyema, gentamicin, serratia marcescens

## Abstract

A 57-year-old male presented with severely altered mental status in the setting of diabetic ketoacidosis. Neuroimaging revealed two intracranial masses. Days following surgical resection of an olfactory groove meningioma, the patient developed *Serratia marcescens* bacteremia along with an enlarging epidural and subgaleal fluid collection. Subgaleal fluid aspiration was also positive. The patient later returned to the operating room for wound washout where purulent collections were discovered in the subgaleal, epidural, and left subdural spaces. The wound was evacuated and the bone flap was thoroughly cleansed with betadine and soaked in peroxide prior to replacement. Four drains were placed (two subgaleal and two epidural) with two serving as inlets and two as outlets. Continuous irrigation of the subgaleal and epidural spaces with gentamicin solution was performed for five days. The bone flap was successfully salvaged and the patient was discharged from inpatient rehab three weeks following washout.

## Introduction

Although relatively rare, the incidence of bone flap infection following craniotomy is reported to range from one to 11%. Known risk factors for such infections include: prior radiation therapy, presence of cerebrospinal fluid leak, duration of surgery over four hours, procedures involving the nasal sinuses or skull base, and emergency surgeries [1-3]. When infection following craniotomy does occur, the traditional standard of care is to surgically discard the bone flap. Unfortunately, this method subjects the patient to months of cosmetic deformity, an unprotected brain, and ultimately, delayed cranioplasty (with potential need for more procedures). Therefore, whenever possible, salvage of the native bone flap is the preferred option [2-4].

We describe a case of post-craniotomy infection treated with thorough cleansing of the bone flap, evacuation of purulent material along with debridement, and continuous irrigation with saline containing gentamicin. This technique resulted in successful salvage of the bone flap and patient recovery.

## Case presentation

**Figure 1:**
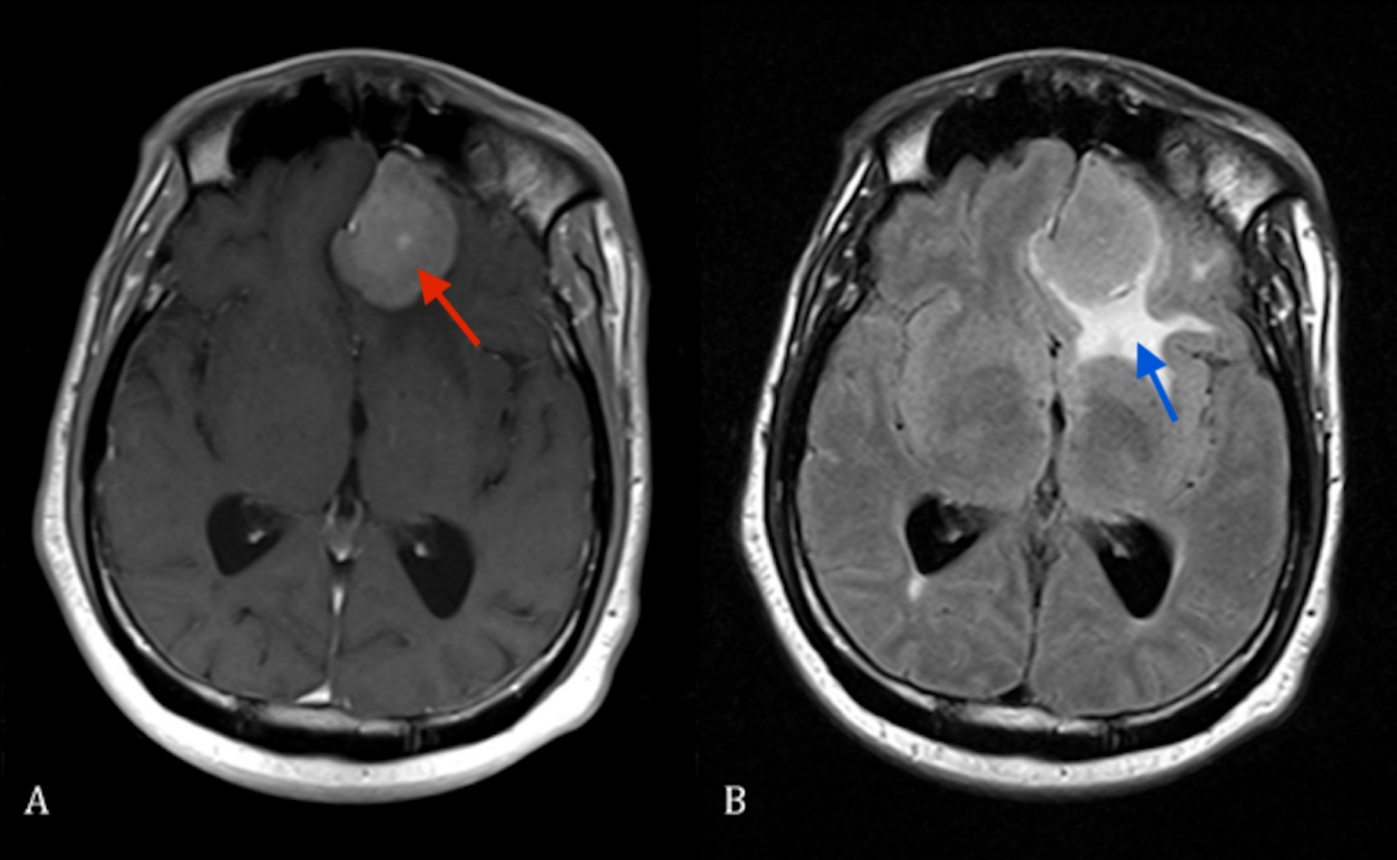
(A) Axial T1 MRI of brain with contrast showing left-sided olfactory groove meningioma (red arrow). (B) Axial FLAIR MRI of brain showing left-sided olfactory groove meningioma and surrounding edema (blue arrow). FLAIR: Fluid-attenuated inversion recovery; MRI: Magnetic resonance imaging.

Our patient, a 57-year-old African-American male with history of cerebral palsy, presented with severely altered mental status and alcohol intoxication in the setting of diabetic ketoacidosis. His Glasgow Coma Scale (GCS) score on admission was three. Computerized tomography (CT) scan and subsequent magnetic resonance imaging (MRI) revealed a left olfactory groove mass with surrounding hypodensity concerning for vasogenic edema (Figure 1) as well as a right petroclival mass.

**Figure 2:**
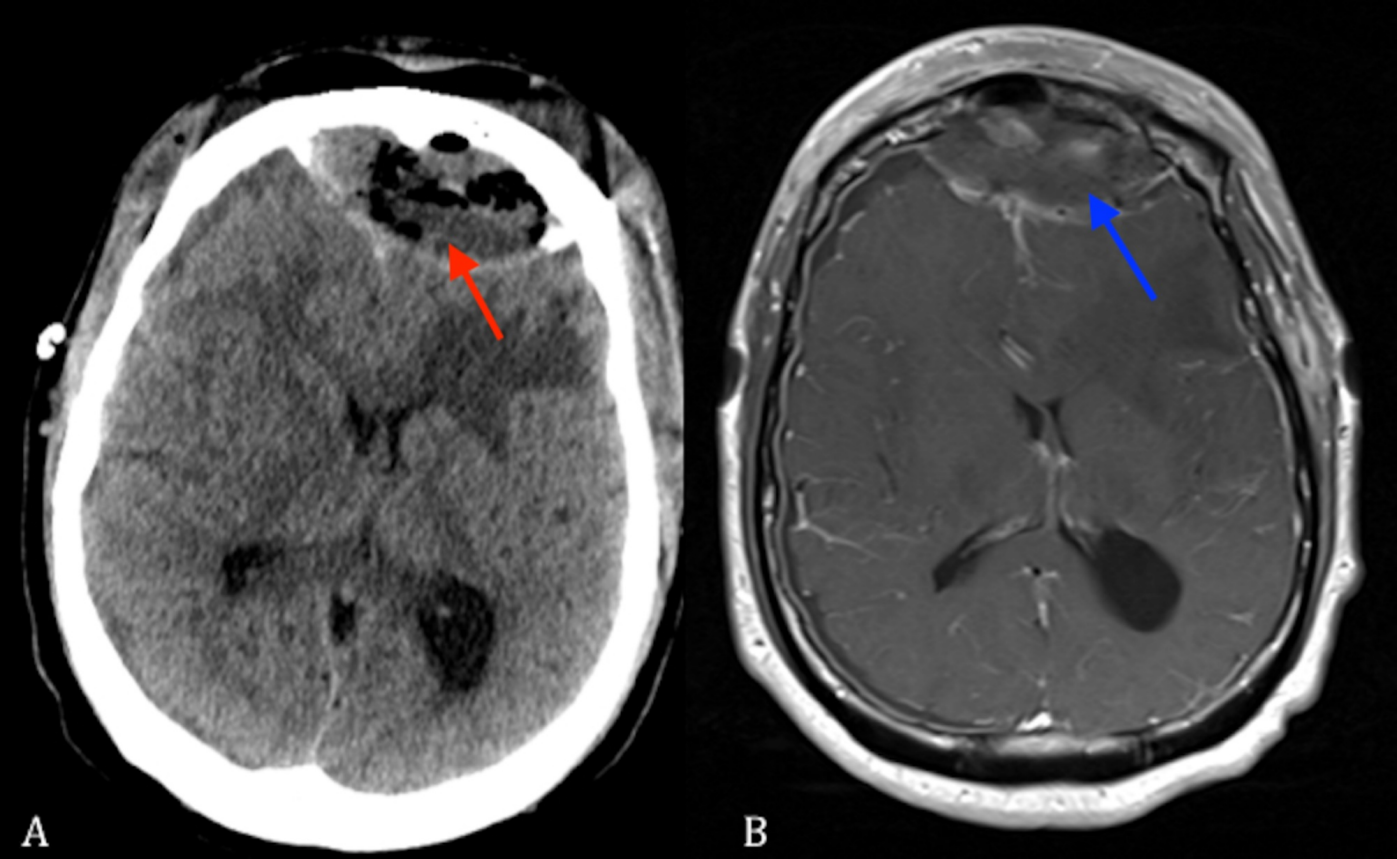
(A) Axial non-contrast CT scan showing a frontal fluid collection under the craniotomy bone flap (red arrow). (B) Axial T1 MRI with contrast showing a frontal fluid collection under the craniotomy bone flap (blue arrow). CT: Computed tomography; MRI: Magnetic resonance imaging.

After the patient was treated medically for his diabetic ketoacidosis and intoxication, he underwent bifrontal craniotomy and resection of the left olfactory groove mass, cranialization of the frontal sinus, as well as pericranial flap placement. Postoperatively, the patient was treated with antibiotics and a dexamethasone taper. An immediate post-operative MRI revealed good resection and expected post-operative changes. The patient did well and remained afebrile, but it was noted that he had increased swelling at his operative site. He developed a markedly elevated leukocytosis on postoperative day (POD) three. Urinalysis and chest radiographs at that time were not concerning for infections. However, blood cultures drawn grew *Serratia marcescens* and the patient was initiated on intravenous meropenem 2 grams every eight hours. Repeat CT imaging obtained on POD 10 revealed an enlarging epidural fluid collection as well as a new subgaleal fluid collection as shown in Figure 2.

Subgaleal fluid aspiration taken at the time grew *S. marcescens* and the patient was taken back to the operating room for wound washout. Antibiotic sensitivity analysis performed on *S. marcescens* revealed a meropenem minimum inhibitory concentration (MIC) of </= 0.25 mcg/mL and a gentamicin MIC of </= 1 mcg/mL.

In the operating room, immediately upon opening the galea, a copious amount of pink-colored purulent material began to spill from the wound. The bone flap was then removed and a collection of purulent material was found in the epidural space. A small subdural empyema was also discovered and subsequently removed. The wounds were irrigated liberally with saline and bacitracin solution. Any abnormal appearing tissue was removed with debridement until there was no evidence of infection.

**Figure 3:**
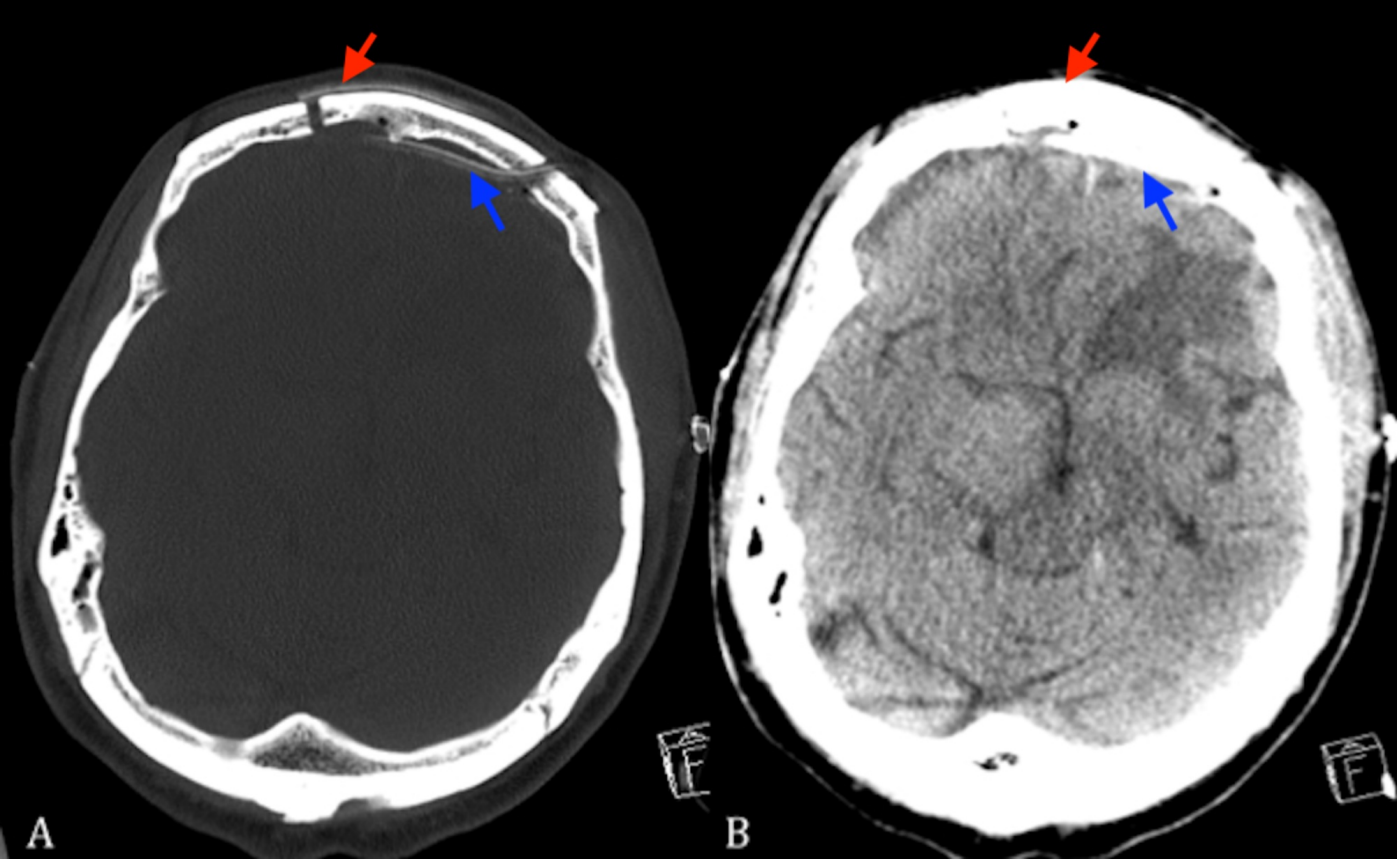
(A) Bone windowed axial non-contrast head CT. (B) Brain windowed axial non-contrasted head CT scan showing placement of subgaleal (red arrow) and epidural catheters (blue arrow). CT: Computed tomography

The bone flap was then extensively cleansed using betadine scrub and was soaked in half-strength peroxide prior to replacement. A total of four drains were placed (two epidural and two subgaleal) with one of each pair serving as inlets and the others as outlets connected to vacuum drains as shown in Figure 3.

Saline solution containing preservative-free gentamicin at a concentration of 20 mcg/mL was continuously infused through the drains at a rate of 10 mL/hour/drain for a period of five days. The patient tolerated the treatment well and was discharged from inpatient rehab three weeks following washout. The patient completed an eight-week course of intravenous meropenem followed by oral levofloxacin for a total of six months of antibiotic coverage.

**Figure 4:**
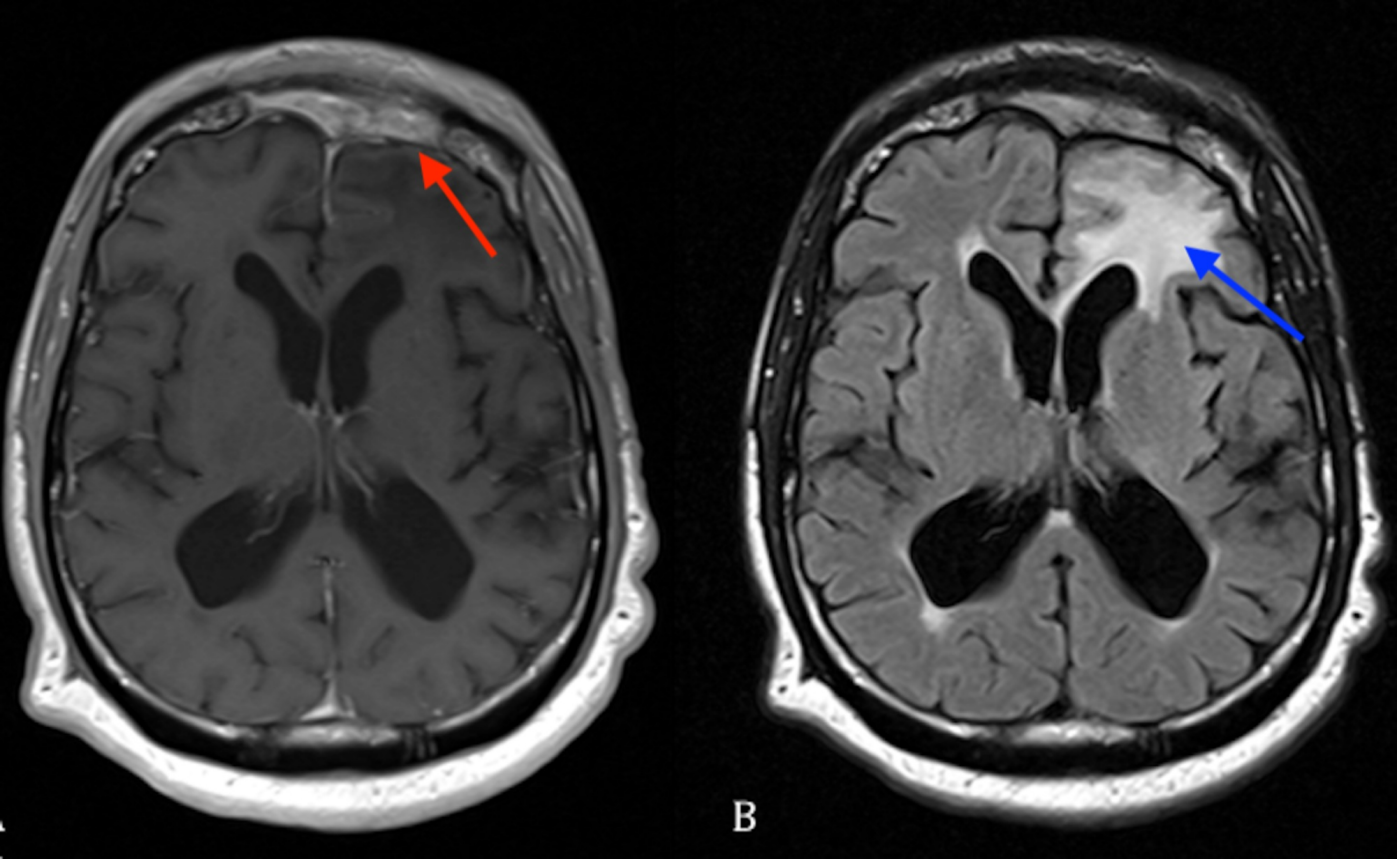
(A) Axial T1 MRI with contrast showing resolution of infection (red arrow). (B) Axial FLAIR MRI showing resolution of infection with some residual flair changes (blue arrow). FLAIR: Fluid-attenuated inversion recovery; MRI: Magnetic resonance imaging.

At five-month follow-up, a repeat MRI demonstrated no signs of infection as shown in Figure 4. The patient had completely returned to his neurologic baseline and received radiation therapy for the remaining meningioma.

## Discussion

Bone flap infection following craniotomy is a relatively rare, but troublesome complication for the patient. The literature reports the incidence of post-craniotomy infection to range from one to 11% [1-3]. Several studies have identified risk factors predisposing patients to post-craniotomy infection. Known procedure-related risk factors include: prior radiation therapy, presence of cerebrospinal fluid leak, duration of surgery longer than four hours, early reoperation, procedures involving the nasal sinuses or skull base, and emergency surgeries. Patient-related risk factors include: GCS score less than 10, American Society of Anesthesiologists (ASA) score greater than two, recent antibiotic use, and recent neurosurgical procedures prior to craniotomy [5-6].

Opinion regarding management of post-craniotomy infection varies. Traditionally, the standard management consists of operative debridement followed by disposal of the bone flap with delayed cranioplasty [1-3]. While proven to be a safe and effective intervention for such infections, the process presents many issues for patients as they are left with a cranial defect and lose protection from head trauma for an extended period of time. In some rare incidences the consequence of large cranial defects, known as the “syndrome of the trephined”, can occur [4-7]. Due to these undesirable factors, many have sought to identify alternative techniques directed at salvaging the bone flap.

In 1974, Erickson et al. employed a technique involving suction and irrigation with an antibiotic solution [8]. During the procedure, the wound was opened, and all visible sutures (as well as necrotic tissue) were removed. Interestingly, the bone flap was left in place. Two drains were then placed in the subgaleal space with one as an inlet and the other as an outlet connected to a Hemovac drain. If purulent matter was seen in the epidural space, a second set of Hemovac drains was placed there as well. An antibiotic solution was then infused at a rate of 1-2 liters/day for a period of up to five days. Close monitoring of drain input and output was necessary as clogged output drains place patients at risk for increased intracranial pressure [3]. Of note, all identified and treated pathogens were skin flora.

In 2003, Bruce and Bruce published a successful debridement strategy that resulted in salvage of native bone flaps [3]. The bone flap was re-elevated and all visible suture and hemostatic agents were removed. Any necrotic or purulent debris was also removed. The bone flap was extensively scrubbed with betadine before replacement. Systemic antibiotic therapy was an important adjunct to success. Patients typically received intravenous antibiotics for one to two weeks followed by two to four weeks of oral therapy. The authors reported a very high degree of success, even in patients with many undesirable risk factors for post-craniotomy infections. Of note, the only risk factor that strongly correlated to failure and loss of bone flap was prior procedures involving communication of the nasal sinuses [2,3]. Our patient was continued on antibiotic therapy for a total of six months. This duration of antibiotics was chosen due to our patient's prior bacteremia and severely uncontrolled diabetic state.

Later, Widdel and Winston implemented an approach similar to Bruce and Bruce [3], with the added implementation of scrubbing the bone flap with iodophor or bacitracin. The bone flap was then immersed in either povidone-iodine or bacitracin until reimplantation. This resulted in successful salvage of all 14 bone flaps in their patient series [4].

Despite the use of antibiotic irrigation systems being described as superfluous by Bruce and Bruce [3] based on their findings, the choice to proceed with a gentamicin irrigation was made in our case due to the uncommon gram-negative bacilli isolated from the patient’s cultures and persistent bacteremia with central nervous system (CNS) involvement despite administration of appropriately dosed broad spectrum intravenous antibiotics. Additionally, Bruce and Bruce [3] note that one of their patient complications requiring additional surgery was in a patient who grew virulent gram-negative bacilli.

In 2006, Auguste and McDermott [2] described a “wash-in, wash-out” method that improved upon the suction-irrigation system implemented by Erickson and Chou [8]. Important differences in protocol included mandatory re-elevation of all bone flaps (with subsequent debridement of the flap and soft tissues), irrigation of both the subgaleal and epidural spaces (rather than subgaleal alone), and at least two weeks of intravenous antibiotics followed by three months of oral therapy. Vancomycin was used for irrigation in all cases and the duration of irrigation was typically five days. This method was less cumbersome in terms of preparation and setup and even allowed for the patients to ambulate. The authors recommended close patient observation as impeded output could result in fluid collection and mass effect. Their treatment protocol resulted in the successful preservation of bone flaps in all but one patient in a 12-patient series. Similar methods of continuous irrigation have even proven useful in the treatment of subdural empyema [9,10]. In all cases vancomycin irrigation was used as the majority of organisms isolated from operative cultures were predominantly skin flora. It is less common for gram-negative bacilli to complicate surgery as opposed to skin organisms such as *Staphylococci* or *Propionibacterium*. Gentamicin is an aminoglycoside antibiotic with broad spectrum coverage of aerobic gram-negative bacteria. Very low cerebrospinal fluid concentrations of the drug are achieved in patients without inflamed meningitis leading to alternative administration routes for the treatment of CNS infections [11,12]. Utilizing a localized route of administration minimizes systemic absorption of the drug and can avoid common adverse effects. The use of a 10 mL/hour infusion rate decreases the risk for complications, such as brain herniation from increased fluid volume, while keeping the gentamicin concentration well above the organism’s reported MIC. It is impossible to measure standard peak and trough concentrations when administering gentamicin by these means. By providing a concentration of 20 mcg/mL of drug locally at an infusion rate of 10 mL/hour, we ensured that the amount of drug present in the subgaleal space stayed above the organism's reported MIC of 1 mcg/mL and obtained a peak/MIC ratio > 10. This localized approach to treatment has been documented in the literature with intraventricular gentamicin administration for the treatment of gram-negative device infections [13-16].

By combining the meticulous surgical techniques described by Bruce and Bruce [3] and Widdel and Winston [4] with the newer “wash-in, wash-out” irrigation system explained by Auguste and McDermott [2], we successfully treated the patient’s infection while preserving the bone flap with no further complications. The patient was closely monitored and could ambulate during the five days of irrigation.

Similarly to our case, is a report published by Wada et al. describing a patient with panparanasal sinusitis, destruction of the frontal sinus walls, and subdural fluid collection resulting in a wide decompressive craniectomy in the bilateral frontal and left temporoparietal regions [9]. After irrigation of the subdural space, six drainage tubes were placed and a seven-day course of continuous antibiotic irrigation with a gentamicin solution of 20 mcg/mL in saline was administered at a flow rate of 10 mL/hr. The patient grew pan-sensitive *Streptococcus milleri*, and the patient’s infectious signs and symptoms resolved quickly without complications to the drainage system. Extrapolating the safety data from Wada et al. [9], we used a similar dose of gentamicin and rate of infusion over a five-day course with success. Our patient had a full recovery and is back at his baseline.

## Conclusions

The methods described above challenge the standard treatment of discarding bone flaps in the setting of post-craniotomy infection. Although the referenced studies contain a relatively small number of patients, they demonstrate that contaminated bone flaps can be salvaged, thus sparing patients undesirable consequences while awaiting cranioplasty. Certainly, the decision to pursue aggressive measures to preserve bone flaps should be based upon individual patient characteristics. When indicated, physicians following similar methods to ours should have high expectations of success. A gentamicin irrigation system is a valuable added therapy for gram-negative infections not responding to broad spectrum intravenous antibiotic therapy.
